# Muscle-Driven Total Knee Replacement Stability with Virtual Ligaments

**DOI:** 10.3390/bioengineering12020112

**Published:** 2025-01-25

**Authors:** Alexandre Galley, Emma Donnelly, Ilya Borukhov, Brent Lanting, Ryan Willing

**Affiliations:** 1 Biomechanical Engineering Research Laboratory, Department of Mechanical and Materials Engineering, Western University, 1151 Richmond St., London, ON N6A 3K7, Canada; agalley@uwo.ca; 2 Biomechanical Engineering Research Laboratory, School of Biomedical Engineering, Western University, 1151 Richmond St., London, ON N6A 3K7, Canada; edonne@uwo.ca; 3 Joint Replacement, Department of Advanced Technology, Stryker Corp., 325 Corporate Dr, Mahwah, NJ 07430, USA; ilya.borukhov@stryker.com; 4 Department of Orthopaedic Surgery, University Hospital, Western University, 1151 Richmond St., London, ON N6A 3K7, Canada; brent.lanting@lhsc.on.ca

**Keywords:** total knee replacement, joint motion simulator, joint kinematics, joint laxity

## Abstract

Knee joint stability comprises passive (ligaments), active (muscles), and static (articular congruency) contributors. The stability of total knee replacement (TKR) implants can be assessed pre-clinically using joint motion simulators. However, contemporary testing methods with these platforms do not accurately reproduce the biomechanical contributions of passive stabilizers, active stabilizers, or both. A key component of joint stability is therefore missing from laxity tests. A recently developed muscle actuator system (MAS) pairs the quadriceps-driven motion capabilities of an Oxford knee simulator with the prescribed displacements and laxity testing methods of a VIVO robotic knee testing system, which also includes virtual ligament capabilities. Using a TKR-embedded non-cadaveric joint analogue, TKR with two different virtual ligament models were compared to TKR with no active ligaments. Laxity limits were then obtained for both developed models using the conventional style of laxity testing (the VIVO’s force/displacement control) and compared with results obtained under similar conditions with the MAS (gravity-dependent muscle control). Differences in joint control methods identified the need for muscle forces providing active joint stability, while differences in the effects of the virtual ligament models identified the importance of physiological representations of collateral ligaments during testing.

## 1. Introduction and Background

For patients with end-stage knee osteoarthritis, total knee replacement (TKR) is an effective treatment for alleviating knee pain and restoring mobility [1,2], with studies reporting survivorship as high as 90–95% after 10 [3,4] or 20 years [5]. However, as many as 1 in 5 patients report dissatisfaction with the surgical outcome [6,7,8]. Some report feeling that their knee may buckle under load; a condition known as joint instability [4,9]. This is widely believed to be caused by excessive joint laxity or inadequate constraint at some flexion angles. Revision surgery that modifies the implanted components’ position, size, or design may be necessary in more severe cases [10]. Joint instability is responsible for up to 20% of early TKR revisions [11,12].

Pre-clinical tests can quantify prosthesis constraint [13], yet the propensity of instability suggests that the functional stability of the knee post-TKR is not fully understood, and new testing techniques may be required. Forces that restrain and stabilize the knee are provided by ligaments, muscles, and the shape of the implant (congruent condylar articulations), which becomes more stable as the ratio of compressive versus shear forces increases. Unfortunately, contemporary pre-clinical tests do not accurately reproduce the biomechanical contributions of ligaments and the muscles crossing the joint, the latter of which being one of the largest contributors to joint compressive forces. This is largely due to the limited capabilities of available prosthesis testing systems, which generally cannot simulate realistic ligaments or muscle forces during laxity tests.

Recently, a robotic knee testing system (RKTS) capable of performing TKR laxity/constraint tests has been introduced with the ability to represent the biomechanical contributions of user-defined virtual ligaments acting around a knee (VIVO, Advanced Mechanical Technologies Inc, Watertown, MA, USA, https://www.amti.biz/product/vivo-simulator/, URL accessed on 18 January 2025). This has enabled new parametric studies that varied a representative ligament model to examine how different TKR balancing strategies affect behavior [14] or ligament models tuned to match post-TKR behaviors of real cadaveric specimens [15]. Moreover, we recently reported on our muscle actuator system (MAS), which adds muscle-tensioning capabilities to the VIVO and has been used to perform gravity-dependent, quadriceps-controlled squat simulations [16]. This system is therefore technically capable of measuring joint laxity with both simulated passive and active contributors to joint stability (ligaments and muscles, respectively). How such measurements differ from laxity measurements obtained using conventional techniques has not been reported. Thus, the current study uses a TKR phantom knee and our paired VIVO-MAS platform to investigate the influence of varying simulated ligamentous constraints and joint loading (with or without muscle forces) on knee joint kinematics and joint laxity.

## 2. Methods

**Figure 1:**
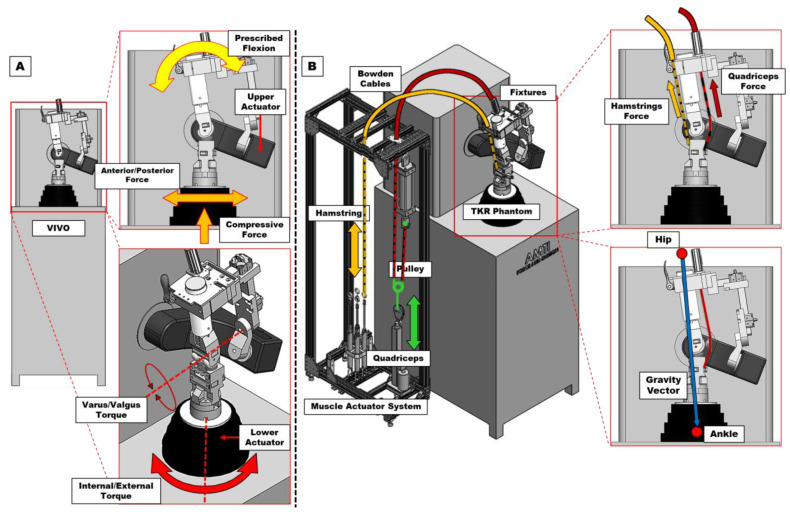
Simplified three-dimensional (3D) models of the joint motion simulator systems. (**A**) The VIVO with custom fixtures mounted to the upper actuator. Laxity studies use the VIVO’s upper and lower actuators to hold the phantom knee in position and apply forces and torques in the anterior–posterior (AP), internal–external (IE), and varus–valgus (VV) directions. (**B**) The muscle actuator system (MAS) with actuators capable of providing quadriceps and hamstrings muscle forces. A gravity vector acts from simulated hip-to-ankle in approximation of a squatting motion.

For the purposes of this study, the mounting platform and conventional RKTS capabilities were denoted as the VIVO (Figure 1A), as were all related tests and outcomes. Similarly, all components responsible for the combined platform’s muscle control capabilities were denoted as the MAS (Figure 1B), as were all tests conducted with flexion/extension motions driven in gravity-dependent muscle control.

### 2.1. Phantom Joints and TKR Implants

**Figure 2:**
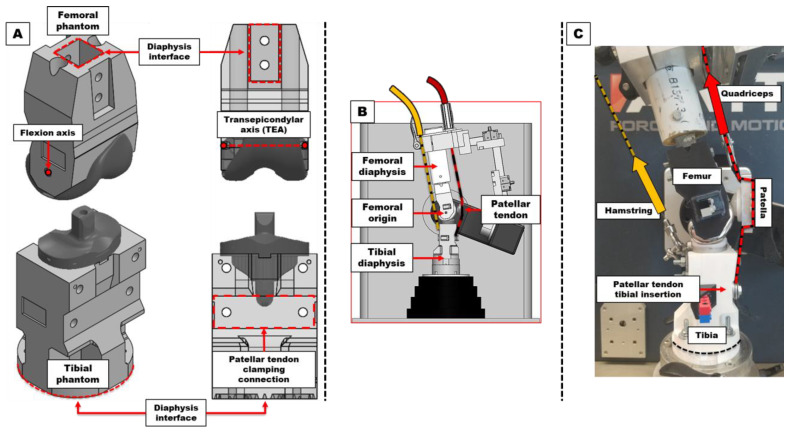
Diagrams of a TKR-embedded phantom knee. (**A**) Three-dimensionally printed distal femur and proximal tibia phantom components. Selected TKR implants were embedded within open internal geometries matching the implants’ non-articular surfaces. (**B**) The phantom’s femoral and tibial diaphyses were clamped within the platform’s femoral and tibial fixtures. The femoral origin (flexion axis of the femur) was aligned with the VIVO’s flexion axis. (**C**) Physical components of the TKR phantom mounted to the femoral and tibial fixtures.

Experiments were performed with a TKR phantom knee. Cylindrical femoral and tibial fixation points (each a simplification of a femoral or tibial diaphysis) were attached to 3D-printed analogues of the distal femur and proximal tibia (Figure 2A). Posterior-stabilized (Triathlon, PS, Size 4, Left, Stryker Orthopaedics, Mahwah, NJ, USA) TKR implants were embedded into these components. The proximal tibia’s posterior surface included two attachments to simulate the medial and lateral tibial condyle insertions of the hamstring muscles [17]. The phantom also included a Kevlar^®^ strap extensor mechanism (quadriceps and patellar tendons) and an aluminum mount for a 9 mm thick patellar button (Triathlon X3 Symmetric patella, 31 mm × 9 mm, Stryker Orthopaedics, Mahwah, NJ, USA). The length of the phantom’s patellar tendon, defined as the distance between the most inferior point on the femoral condyle and the inferior margin of the patella button, was set to 37 mm [18]. Prior to testing, all articular surfaces of the TKR implant components were lubricated with a silicone-based lubricant.

### 2.2. Simulating Joint Motion

The phantom’s femoral and tibial fixation points were mounted onto the platform using the fixtures and setup shown in Figure 2B. The external sheaths of the MAS’ two Bowden cables were attached to the platform’s femoral fixture. Their inner cables were routed through the fixture and connected to the phantom’s simulated quadriceps tendon and hamstring attachments (Figure 2C). For the quadriceps force line-of-action, a Q-angle of 11.5° was applied. To replicate gravity forces (Figure 1B), virtual hip and ankle coordinate points were calculated based on assumed positions relative to the extended knee (475 mm superior and 455 mm inferior, respectively) [19].

### 2.3. Virtual Ligament Models

The VIVO incorporates the force contributions of virtual one-dimensional (1D) point-to-point springs with a piecewise but continuous nonlinear force versus elongation response [20,21], such that:

(1) $$f=\left\{\;\begin{array}{l} (\frac{1}{4}(k\varepsilon^{2}\;))/\varepsilon_{l}\;,\;\;\;\;\;\;\;0\leq \varepsilon \leq 2\varepsilon_{l}\\ k(\varepsilon -\varepsilon_{l}),\;\;\;\;\;\;\;\;\;\;\;\;\;\;\;\varepsilon >2\varepsilon_{l}\\ 0,\;\;\;\;\;\;\;\;\;\;\;\;\;\;\;\;\;\;\;\;\;\;\;\;\;\;\;\;\varepsilon <0\; \end{array}\right.$$

where f is the tensile force in a virtual ligament or spring, and k is the ligament stiffness. Force was assumed to be linear for strains higher than $2\varepsilon_{l}$ ($\varepsilon_{l}$ is a strain constant assumed to be 0.03) [20,21]. Ligament strain (ε) was calculated from its length l (linear distance between user-defined insertion points) relative to its reference length $l_{r}$ and a user-defined reference strain ($\varepsilon_{r}$), both of which refer to the length and strain of a ligament in the joint’s initial reference position (in full extension).

(2) $$\varepsilon =\frac{l}{l_{r}}\left(1+\varepsilon_{r}\right)-1$$

Virtual ligaments were created, comprising single-bundle virtual representations of the superficial medial collateral ligament (MCL) and lateral collateral ligament (LCL). Virtual anterior and posterior cruciate ligaments (ACL, PCL) and the deep MCL (dMCL) were not included, as they are released for PS-TKR [22]. The length of the virtual MCL and LCL were calculated using measurement ratios and scaling factors adapted from Otake et al. [23], and stiffness values were derived from Bloemker et al. [24]. Reference strains were calculated based on average medial–lateral (ML) compartment forces derived from K Cho et al. [25], adjusted such that the MCL and LCL tensons were each approximately 100 N. The resulting ligament properties are shown in Table 1.

**Figure 3:**
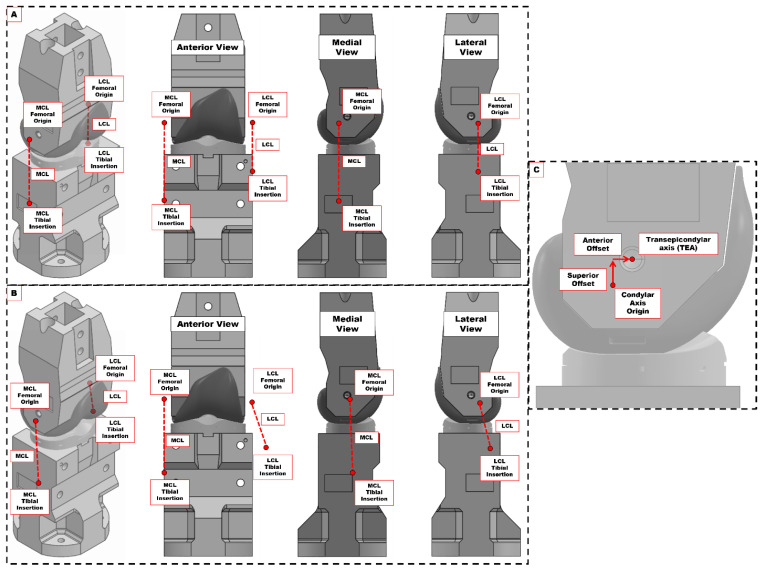
Femoral and tibial attachments of the virtual MCL and LCL on the TKR phantom knee. (**A**) Isometric ligament model. (**B**) Physiological ligament model. (**C**) Measuring offsets of the ligament attachments points for the virtual ligaments with respect to the defined transepicondylar axis (TEA). Example shown for the isometric model, where femoral attachments of the virtual MCL and LCL are in line with the condylar axis origin.

Ligament insertion points were modified to create *isometric* and *physiological* virtual ligament models. The *isometric* model (Figure 3A) was designed to represent a “well balanced” TKR knee, or the positions of synthetic collateral ligaments during non-cadaveric in vitro tests [26,27,28]. The femoral attachments of the MCL and LCL were positioned along the femoral condylar axis, which was determined by a sphere-fit of the medial and lateral TKR femoral condyles, at the transepicondylar widths adapted from Servien et al. [29]. The tibial insertions were then positioned directly inferior of the femoral insertions by the defined MCL and LCL lengths (68.16 mm and 44.75 mm, respectively) with no deviation in the sagittal or coronal planes.

Due to small errors in phantom positioning and alignment onto the VIVO and machine compliance [30,31], ligament forces with the *isometric* model were not constant, and the mean MCL and LCL forces across the entire flexion range of motion were 89 ± 18 N and 102 ± 23 N, respectively. The contributions of virtual ligaments during baseline testing are summarized in Appendix A.1.

For the *physiological* model, the ligament insertions were positioned to simulate those of the (superficial) MCL and LCL observed in cadaveric knees. The femoral insertions were positioned using landmarks, measurement ratios, and scaling factors, again from Otake et al. [23], adapted for use with the 3D model of the TKR femoral implant. The tibial insertion of the MCL was also positioned using this adapted method. In this model, the lateral position of the LCL’s tibial insertion was at the approximated position of the fibular head, based on the work of Guess et al. [32]. In both models, the virtual coordinates of the ligaments’ femoral and tibial attachments were calculated with respect to the TKR phantom’s defined transepicondylar axis (TEA) (Figure 3C).

### 2.4. Test Conditions

The zero position of all degrees of freedom (DoFs) was defined as the joint’s alignment when subjected to a compressive force 200 N at 0° extension, with all other DoFs unrestrained [33] and no virtual ligament models. The baseline loading scenario comprised continuous cyclical flexion and extension motions between 15° and 90°, with each motion cycle completed over a period of 120 seconds. For each test performed, the mean and standard deviation of data at specific angles were calculated across n = 3 trials, with each trial based on the average of n = 3 flexion/extension motions. Tibiofemoral (TF) kinematics were measured using optical trackers (Optotrak Certus, NDI, Waterloo, ON, Canada; marker accuracy of ± 0.1 mm) mounted onto the phantom’s distal femur and proximal tibia. Joint kinematics were described using the Grood and Suntay coordinate conventions [34,35].

Biomechanical tests were first performed using conventional RKTS (VIVO) force/displacement methods. The joint was reduced using a 75 N compressive load applied along the tibia’s longitudinal axis. This compressive load was selected to be compatible with the muscle-driven tests that followed (described below). Flexion/extension was prescribed in displacement–control using imported motion waveforms. Forces or moments in all other DoFs were maintained at 0 N/0 Nm in force–control when measuring neutral path kinematics. To measure joint laxity, tests were repeated with superimposed anterior–posterior forces (AP, ± 80 N), internal–external torques (IE, ± 4 Nm), or varus–valgus torques (VV, ± 8 Nm).

The outlined series of tests were then repeated with flexion driven in muscle control (MAS tests). Flexion torque was generated by simulating 75 N of gravity acting hip-to-ankle. This was 50% higher than the minimum ankle/ground reaction force recommended for physiological kinematics during muscle-driven motion [36,37]. The VIVO’s control system maintained equality between gravity-generated flexion torques and quadriceps-driven extension torques by adjusting the flexion angle. A constant force of 23 N [19] was applied at the hamstring to reduce excessive anterior translations and tibial rotations, increasing the knee’s natural active stability [38]. The maximum flexion moments due to virtual gravity recorded during baseline motion tests were 25.0 Nm (no ligaments), 28.8 Nm (*isometric* model), and 32.1 Nm (*physiological* model). These were similar to the peak flexion moments reported by conventional in vitro testing conditions with Oxford-style knee simulators [26].

### 2.5. Data Recording, Processing, and Analysis

Kinematics and muscle forces were collected by the MAS’ custom MATLAB program at 10 Hz, then smoothed using a 2nd-order low-pass Butterworth filter in a separate custom MATLAB program. All data were then resampled at 5° increments of TF flexion and extension, and the mean of flexion and extension at these respective increments were calculated across trials.

Neutral path TF kinematics (AP, IE, and VV) of the VIVO baseline were first compared to those obtained from tests conducted in active muscle control (MAS tests). For both methods of joint control, data were then compared to those obtained by tests conducted with the individual *isometric* and *physiological* ligament models, thus implementing passive stability contributors. The mean MCL and LCL forces calculated for either ligament model, and their effects on quadriceps forces, are described in Appendix A.2.

With one of the ligament models always active, laxity tests were then conducted to evaluate the same DoF at the same increments of flexion. The effects of the joint control methods were compared across the virtual ligament models. Similarly, the effects of these models were compared across the control methods. In addition to the laxity measure for each test, the sum or range of opposing laxity limits was calculated at specific joint flexion angles of 15°, 45°, and 90°. These were denoted as the laxity envelope (or envelope of laxity, EoL = Upper Limit + Lower Limit). Data were not obtained for anterior laxity tests during conventional RKTS (VIVO) testing methods due to TF joint dislocation when the anterior load was applied. All laxity limits of the TF joint, for any test, were reported relative to the kinematic measurement obtained during associated baseline tests.

For the n = 3 trials conducted per test, an analysis of variance (univariate ANOVA) was used to determine if the flexion angle (15–90° in 15° increments), joint control (MAS, VIVO), and virtual ligament models (no-ligament baseline, *isometric*, *physiological*) had significant effects on TF kinematics and the respective laxity limits (three-way ANOVA). A significance level (α) of 0.05 was used (*p* < 0.05). Due to the small sample size of this study, the resulting *p*-values were not corrected for the multiple comparisons considered for each measure. Post hoc comparisons of means and pairwise comparisons were conducted at specific flexion angles if either other independent variable (joint control methods or virtual ligament model) or their interactions with the flexion angle were determined to be significant.

## 3. Results

### 3.1. Neutral Path Kinematics

#### 3.1.1. Effect of Joint Control Methods

**Figure 4:**
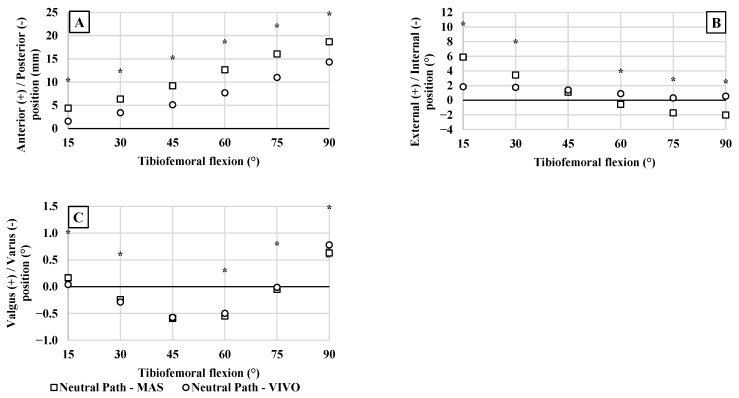
Average neutral path kinematics comparing joint control methods. Results are shown for (**A**) AP translations, (**B**) IE rotations, and (**C**) VV rotations. Neutral paths of motion during MAS tests that differed significantly from motion during VIVO tests are denoted with an asterisk (*).

The neutral path kinematics of the no-ligament baseline were shown to be significantly influenced by using muscle control (MAS) versus conventional (VIVO) loading (*p* < 0.001 for AP, IE, and VV) (Figure 4). This was most prominent for AP tibial translations, having a significant effect at all flexion angles (*p* < 0.001). There were also significant effects on IE (*p* < 0.001) and VV (*p* ≤ 0.019) kinematics, except at 45° in both cases.

#### 3.1.2. Effect of Virtual Ligaments

**Figure 5:**
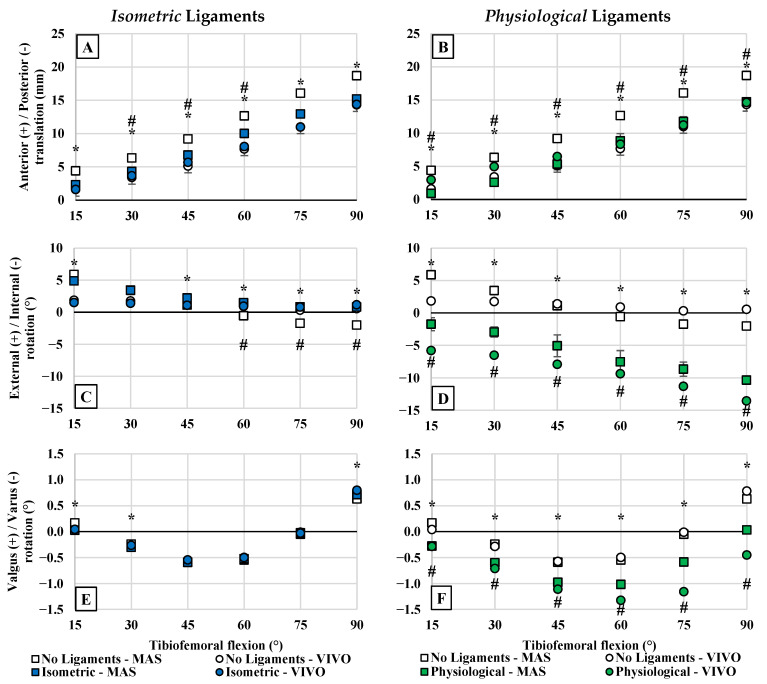
Average neutral path kinematics comparing the isometric and physiological ligament models to the no-ligament baseline. Results are shown for AP (**A**: isometric, **B**: physiological), IE (**C**,**D**), and VV (**E**,**F**) kinematics. Neutral paths of motion during MAS tests with virtual ligaments that differed significantly from the no-ligament baseline are denoted with an asterisk (*). Neutral paths of motion during VIVO tests with virtual ligaments that differed significantly from the no-ligament baseline are denoted with a number/pound sign (#).

Implementing the *isometric* ligament model had a significant effect on kinematics (Figure 5) (*p* < 0.001 for AP, IE, VV). During the conventional RKTS tests (VIVO tests), the effects of the *isometric* ligament model were only significant between 30–60° for AP translations (*p* = 0.02), and between 60–90° for IE rotations (*p* = 0.031). During the MAS tests with the *isometric* ligaments, the effects of the ligament model were significant at all flexion angles (*p* < 0.001) for AP translations. For IE rotations, these effects were significant at all flexion angles (*p* < 0.001) except 30°.

Implementing the *physiological* virtual ligament model had a more prominent effect on TF kinematics. Its effects were significant for all measured DoFs and at all flexion angles (*p* < 0.001 for MAS, *p* ≤ 0.023 for VIVO tests). During the VIVO tests, the *physiological* model caused a mean anterior translation of 0.9 ± 0.6 mm (peak of 1.5 mm at 30°) relative to the no-ligament baseline. In comparison, the same model caused the tibia to be positioned more posteriorly during the MAS tests (mean −3.9 ± 0.2 mm, peak of −4.3 mm at 75°). Considering IE kinematics, the *physiological* model caused greater internal tibial rotation by a mean of −9.7 ± 2.5° (peak of −13.7° at 90°) during the VIVO tests and a mean of −7.0 ± 0.8° (peak of −8.3° at 90°) during the MAS tests. For VV kinematics, mean varus inclinations were recorded for both the VIVO tests (−0.8 ± 0.4°, peak of −1.2° at 90°) and MAS tests (−0.5 ± 0.1°, peak of −0.6° at 90°).

### 3.2. Laxity Testing

**Figure 6:**
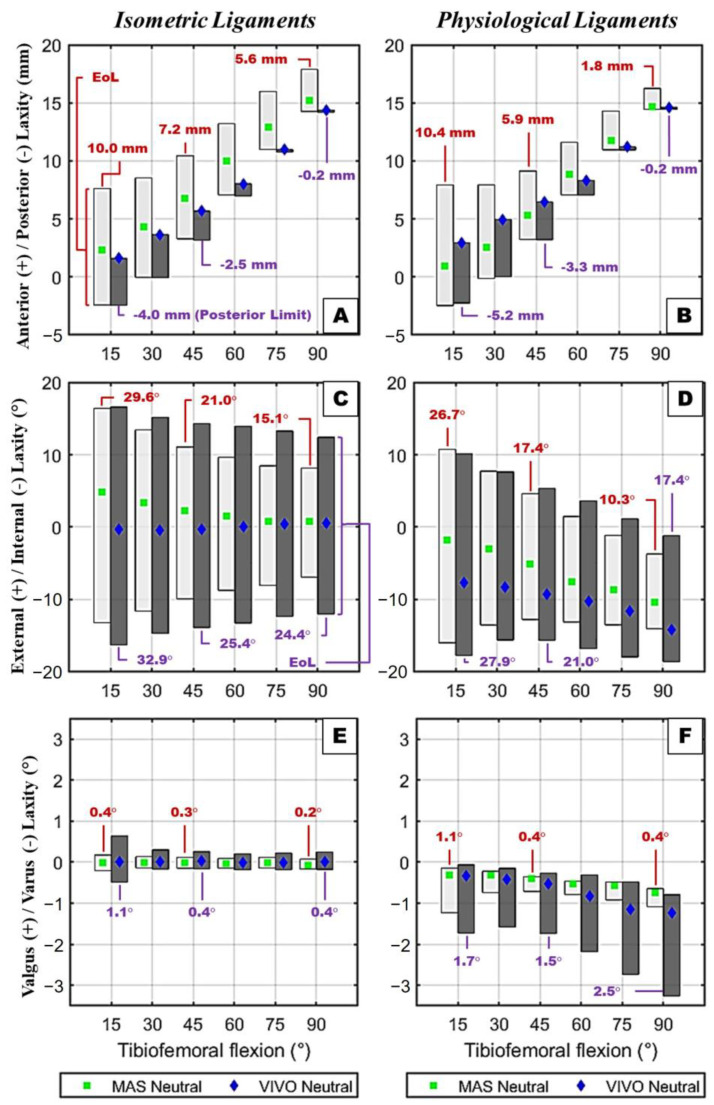
Tibiofemoral kinematic laxity limits with the isometric and physiological ligament models. Results are shown for AP (**A**: isometric, **B**: physiological), IE (**C**,**D**), and VV (**E**,**F**) kinematics. Laxity envelope (EoL) values are shown for MAS (red) and VIVO (purple) tests at 15°, 45°, and 90°.

For both ligament models, the total AP laxity envelopes decreased as the flexion angle increased (Figure 6A,B). Interestingly, despite the neutral flexion AP kinematics being sensitive to how the joint was loaded, the measured posterior laxity limits were nearly identical for both the VIVO and MAS tests. There was greater sensitivity to the ligament models being employed. Unfortunately, due to dislocations when anterior-directed loads were applied, the anterior laxity could not be measured during the VIVO tests.

The IE laxity envelopes decreased with increasing flexion and were generally smaller when the physiological ligament models were used (Figure 6C,D). Also with the physiological ligament model, just as the tibia tended to internally rotate with flexion, the laxity limits drifted internally. More symmetrical behavior was observed with the isometric ligaments. Neither of these behaviors appeared sensitive to how the joints were tested; however, the VIVO tests generally resulted in wider IE laxity envelopes than the MAS tests.

The VV laxity envelopes showed very little sensitivity to the flexion angle, in terms of overall size or bias, when the isometric ligament models were used (Figure 6E). This was true for both testing methods (MAS and VIVO). With the physiological ligament model, the laxity limits were biased varus as the flexion angle increased, which is the same trend as what was observed for the neutral kinematics (Figure 6F). The amount of varus laxity (and overall laxity envelope) for the physiological ligaments was greater during the VIVO tests than during the MAS tests, likely due to the lower compressive forces.

## 4. Discussion and Conclusions

The main objective of this study was to use the MAS to manipulate joint flexion and assess any differences caused by the different forms of joint control during pre-clinical evaluations of TKR laxity when active and passive stability contributors were replicated. The MAS, which combines the muscle control capabilities of an Oxford-style knee simulator with the conventional RKTS joint control methods of a VIVO, was used to manipulate joint flexion under gravity-dependent quadriceps control. Tibiofemoral kinematics were recorded and compared to the results obtained with the RKTS control methods.

The neutral path kinematics of the PS TKR without ligaments were largely as anticipated. There was a gradual anterior displacement of the tibia, consistent with the intended action of the post-cam mechanism. The tibia was consistently more anterior throughout motion when the joint was manipulated with the application of quadriceps forces. The influence of these forces was expected to be dependent on the flexion angle [39]; however, with no soft tissue constraints besides virtual collateral ligaments stabilizing the joint, the anterior offset appeared exaggerated and consistent through the entire flexion arc. Also, when quadriceps forces were applied, the pre-set Q-angle of 11.5° caused an initial external rotation of the tibia in extension. As the knee flexed, the relative internal rotation was caused by the compressive forces through the joint created by the quadriceps. This behavior was supported by in vitro and in silico work [38,39,40,41,42] and by descriptions of intact in vivo knee kinematics [43]. There was a small amount of varus/valgus rotation, consistent across both motion control techniques, which could be attributed to a small misalignment of the femoral component relative to the VIVO.

The addition of ligaments primarily had two effects on neutral path kinematics. Firstly, regardless of the ligament model used, the anterior tibial translation during quadriceps-driven flexion–extension motions was reduced and matched more closely with the AP kinematics of the tests without simulated muscle forces. Secondly, the diagonal trajectories of the medial and lateral collateral ligaments in the physiological model created a net internal torque across the joint, causing the rotation of the tibia to be biased internally. This generally agrees with the natural behavior of the knee [44]. A third effect was a small increase in varus rotation for the physiological ligament model only, caused by greater tension/stiffness of the medial collateral ligament.

The present study introduces an additional method for testing TKR laxity when the joint is manipulated in gravity-dependent quadriceps control, which was previously difficult to conduct with standard knee simulators like the Oxford rig. Laxity limits and EoLs measured during the MAS tests were generally smaller than those measured when flexion was prescribed directly by the VIVO. Simulating the restraints provided by the patellar and quadriceps tendons and implementing a quadriceps force to the patella also prevented tibial dislocation when an anterior laxity force was applied (which otherwise occurred during our conventional laxity tests).

In vitro [33] and in silico [45] RKTS studies that utilized (or modeled) cruciate-retaining and/or cruciate-stabilizing TKR designs (Triathlon, CR and CS, Stryker, Mahwah, NJ, USA) included the full soft tissue structures of the collateral ligaments. In the case of the cited in silico study by Montgomery et al. (with simulated laxity loads of −100 N posterior, ±4 Nm IE, and ±8 Nm VV), multi-bundle ligament models were developed for the MCL, LCL, and PCL. In comparison, this current study modelled the MCL and LCL as single bundles and omitted the PCL. This allowed for simplicity in development and ease in their parametrization as *isometric* and *physiological* models. However, it also created differences in behavior compared to the more realistic multi-bundle virtual ligaments, as did the use of PS TKR implants [15,46]. These findings were supported by an RKTS study by Sekeitto et al. [22], which used a combination of in silico and empirical methods to simulate joint laxity tests of isolated PS TKR implant components on a VIVO RKTS. Sekeitto used a patient-specific ligament model with reference strains derived from the literature. Like this study, they also developed single-bundle collateral ligaments representing the superficial MCL and the LCL. Laxity tests in the posterior (100 N) and varus–valgus (±10 Nm) directions were conducted with a constant 10 N compressive load to reduce the implants. Trends for their reported posterior laxity limits agreed with those of this study for both types of ligament models, for the MAS and VIVO tests alike. In all cases, posterior laxity was greatest during extension and decreased during flexion. This was primarily due to the engagement of the PS TKR implants’ post-cam mechanism as flexion increased. The varus–valgus laxity trends reported in this cited work also agreed with those in the present work for the MAS and VIVO tests and *physiological* ligaments, showing an increase in varus laxity as the knee flexed. Increased varus versus valgus laxity has also been reported in in silico studies based on in vivo kinematics [46]. The reduced EoLs observed in this present study, compared to the cited work by Sekeitto et al., confirmed that implementing muscle forces to control flexion provided a net benefit to TKR stability during pre-clinical laxity tests.

Hunt et al. [11] also made alterations to conventional methods of in vitro laxity testing in an attempt to simulate muscle loading. Extensor forces remained constant as the joint was flexed and extended. Possible explanations for disagreements between these studies and this present work might include the TKR design (Triathlon, CR, Stryker, Mahwah, NJ), cadaveric versus synthetic specimens, and differences in loading protocols. However, the largest discrepancy lies in the fact that this present study’s quadriceps forces varied autonomously to manipulate joint flexion, whereas theirs remained constant throughout. The influence of quadriceps forces on joint motion and its dependence on the joint flexion angle has previously been described [39,40]. In conventional muscle-driven Oxford rig studies, quadriceps forces increase with joint flexion, in turn maintaining a realistic level of active stability [36,37,47]. The application of constant quadriceps forces, as observed in previous attempts to simulate muscle loading during RKTS laxity tests, were therefore not accurately portraying realistic quadriceps forces and further affirmed that implementing motion-driven quadriceps forces, as observed in standard knee simulators, increased TKR joint stability during pre-clinical tests.

Throughout this study, a secondary objective was to compare the effects of two virtual ligament models on joint kinematics, laxity limits, and simulated muscle and virtual soft tissue forces. This study’s virtual ligament design approach was based on adapted scaling ratios, measurement techniques, and established tension force and stiffness parameters from previous computational (in silico) [24,32], in vitro [14,23,25,48], and in vivo [29] studies described in the literature. The ligament insertions of the *isometric* model were based on the less physiologically relevant positions of linear springs used in non-cadaveric in vitro tests [26,27,28]. Compared to the *physiological* model, the *isometric* ligaments resulted in smaller differences with respect to the no-ligament baseline. The kinematics obtained with the *isometric* model were therefore less realistic than those produced with the more clinically relevant *physiological* ligaments. This finding was supported by one article studying the effects of ligament modeling techniques on knee joint kinematics [49]. Compared to more physiological models, they reported that oversimplified elastic representations of ligaments (i.e., with simplified positioning, like the *isometric* ligaments) resulted in predicted kinematics that had fewer similarities with empirical results obtained in vivo.

The effects of implementing virtual ligaments on TF kinematics were most prominent during the MAS tests. When implemented, they opposed the forces applied to the tibia by the MAS quadriceps actuator. No such quadriceps forces were applied during the VIVO tests, and the ligament models had a reduced effect on AP kinematics. This reduction in anterior tibial translation was supported by one study describing the MCL and LCL as secondary restraints to anterior displacements [44], with the results being the greatest when *physiological* ligaments were used. These findings and comparisons have shown that the MAS, which leverages the VIVO’s virtual ligament capabilities, can provide a more effective method of simulating the collaterals than the simplified methods commonly observed in non-cadaveric in vitro studies using Oxford rig knee simulators.

The tested range of motion was less than the full flexion range of a real knee joint; however, similar flexion ranges have been reported by other platforms [36,37,50]. Comparisons to cadaveric studies in the literature were made with the knowledge that although behavioral trends were generally in agreement between the present study and the literature, most of the differences between the studies could be linked to differences between cadaveric knees and this TKR phantom joint. The use of a non-cadaveric phantom joint was beneficial for obtaining consistent testing results and for adjusting independent parameters, like virtual ligaments. This is an advantage over cadaver studies, as it provided us the opportunity to parametrize ligaments with a single knee joint; this is more technically challenging with a single cadaver knee. However, any non-cadaveric model will be limited by its oversimplification of surrounding stabilizing soft tissue structures. This may result in exaggerated motions, increasing the risk of tibial dislocation, as observed in this study. Furthermore, the damping and viscoelastic properties of real soft tissues are not represented in the virtual ligament models or physical extensor mechanism developed in this work.

Muscle-driven joint loading was shown to provide active stability to the knee joint. Implementing physiological virtual ligament models on a TKR phantom knee produced stability trends that generally matched cadaveric studies in the literature, providing relevant passive stability previously missing from non-cadaveric knees. Combined, the three forms of joint stability that exist in vivo are now possible during pre-clinical TKR testing. This can potentially provide a more comprehensive understanding of how stable post-TKR knee joints may behave during different activities in vivo. Laxity assessments during TKR surgery or post-operatively in clinic may not adequately reproduce joint stability during weight-bearing activities. This study has shown that the MAS can not only reproduce joint stability for weight-bearing motions but also parametrize independent variables that cannot be changed in vivo.

## Figures and Tables

**Table 1:** Ligament parameters held constant across this study for both the isometric and physiological ligament models.

Parameter	MCL	LCL
Lengths	68.16 mm	44.75 mm
Ligament stiffness (N)	5500 N	4000 N
Reference strains	0.0354	0.0391
Mean force during *isometric* position adjustments (N)	89 ± 18 N	102 ± 23 N

**Figure A1:**
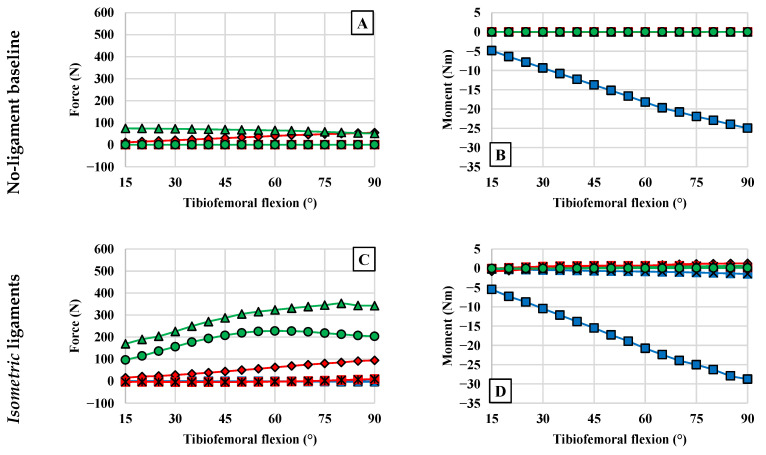

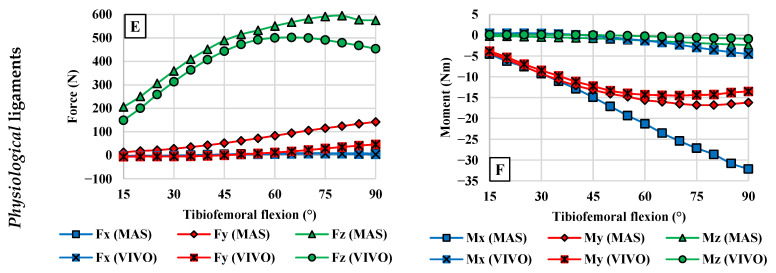
Three-dimensional components of forces and moments recorded by the VIVO’s lower actuator load cell. Plots are shown for MAS and VIVO test results for the no-ligament baseline (**A**: forces, **B**: moments), isometric model (**C**,**D**), and physiological model (**E**,**F**).

**Figure A2:**
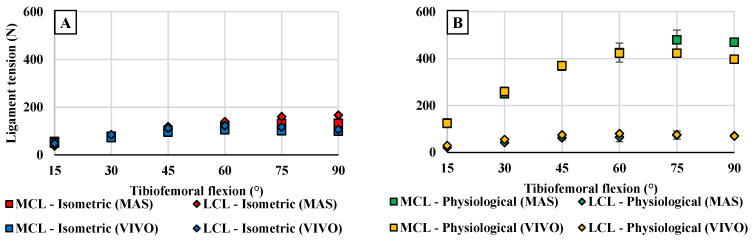
Forces measured during neutral kinematics portion of study. (**A**) MCL and LCL forces during MAS and VIVO tests for the isometric ligament model. (**B**) MCL and LCL forces during MAS and VIVO tests for the physiological ligament model.

## Data Availability

The original contributions presented in this study are included in the article. Further inquiries can be directed to the corresponding author(s).

## Abbreviations

The following abbreviations are used in this manuscript:

3D	Three-dimensional
AMTI	Advanced Mechanical Technology, Inc.
AP	Anterior–posterior
DoF	Degree of freedom
IE	Internal–external
MAS	Muscle actuator system
ML	Medial–lateral
RKTS	Robotic knee testing system
TF	Tibiofemoral
TKR	Total knee replacement
VV	Varus–valgus

## Appendix A

### Appendix A.1. Contributions of Virtual Ligaments During Baseline Testing

In this study, the three cartesian axes (X, Y, Z) were defined as:

- Z-axis: pointing vertically upwards, parallel with gravity.
- X-axis: perpendicular to the Z-axis, pointing towards the right.
- Y-axis: perpendicular to both Z- and X-axes, pointing forward.

Three-dimensional force and moment components created by virtual gravity or ligaments were recorded by the VIVO. This included those generated by the virtual gravity vector applied during the MAS tests. These were broken down by the following convention:

- $F_{z}(M_{z})$: Force along (or moment about) the Z-axis. Superior/inferior forces (internal/external moments)
- $F_{x}(M_{x})$: Force along (or moment about) the X-axis. Medial/lateral forces (flexion/extension moments).
- $F_{y}(M_{y})$: Force along (or moment about) the Y-axis. Anterior/posterior forces (varus/valgus moments).

For all results in this study, those obtained using force/displacement joint control or the conventional style of laxity testing are denoted as VIVO test results, whereas those obtained while manipulating the flexion angle in muscle control are denoted as MAS test results. Force and moment components were measured during the MAS and VIVO tests for the no-ligament baseline and two virtual ligament models (*isometric* and *physiological*) (Figure A1). For the MAS baseline test, the recorded forces show that the virtual gravity vector created superior and anterior forces. As the knee flexed, the superior forces decreased, while the anterior forces increased. At 90° joint flexion, the superior and anterior forces were within ± 5 N of each other. These forces were reflected by a flexion moment that increased with joint flexion. No forces or moments were recorded during the VIVO baseline test, as no virtual forces were implemented.

Forces recorded with the *isometric* ligament model active showed increased superior forces compared to the MAS and VIVO baselines. An increased anterior force was also observed during the MAS tests. The recorded flexion moment was greater in flexion during the MAS tests with the *isometric* model, but other moments (including those recorded during the VIVO tests) were still within ± 2 Nm from their baseline value (0 Nm). Forces recorded with the *physiological* ligament model active showed an even higher increase in superior forces during both the MAS and VIVO tests and anterior forces during the MAS tests. In addition, the moments in the coronal plane (varus/valgus) were initially greater by approximately 5 Nm and increased with joint flexion. A slight flexion moment was observed during the VIVO tests when the knee was flexed.

### Appendix A.2. Mean Ligament Force Measurements and Effect of Ligament Models on Quadriceps Forces

The tension forces of the virtual MCL and LCL did not change considerably between the MAS and VIVO tests. Considering the *isometric* model (Figure A2A), the mean ligament forces were higher during the MAS tests versus the VIVO tests by 17 ± 12 N (MCL) and 18 ± 28 N (LCL). The forces gradually increased with knee flexion for both methods of joint control. Considering the *physiological* model (Figure A2B), MCL forces were higher during the MAS tests versus the VIVO tests by 20 ± 35 N, whereas LCL forces were lower by −8 ± 7 N. Virtual MCL force patterns for the *physiological* ligament model showed a faster and considerably larger increase in force than the *physiological* LCL and both *isometric* ligaments, increasing to as high as 480 ± 42 N during the MAS tests and 423 ± 2 N during the VIVO tests.

Implementing either *isometric* or *physiological* ligament models resulted in lower mean quadriceps forces compared to the no-ligament baseline. A peak force of 696 ± 15 N was observed with the baseline. Implementing the *isometric* model reduced quadriceps forces by a mean −5 ± 8 N, but a similar peak force of 692 ± 11 N was noted. Implementing the *physiological* model reduced quadriceps forces by a mean −24 ± 39 N but caused a slightly higher peak force of 722 ± 51 N.
